# When landmarks are not enough

**DOI:** 10.7554/eLife.87771

**Published:** 2023-04-21

**Authors:** Paul F Hill

**Affiliations:** 1 Department of Psychology, University of Arizona Tucson United States

**Keywords:** spatial navigation, spatial cognition, human aging, landmark, geometry, Human

## Abstract

Including geometric spatial cues in an environment can help reverse the difficulties with spatial navigation experienced by children and older adults.

**Related research article** Bécu M, Sheynikhovich D, Ramanoël S, Tatur G, Ozier-Lafontaine A, Authié CN, Sahel J-A, Arleo A. 2023. Landmark-based spatial navigation across the human lifespan. *eLife* **12**:e81318. doi: 10.7554/eLife.81318.

Our ability to navigate from place to place, which is essential for our wellbeing and independence, declines as we get older (Head and Isom, 2010). Even in healthy individuals, this decline can have a significant impact on quality of life and can foreshadow the onset of Alzheimer’s disease years before the appearance of clinical symptoms (Levine et al., 2020). Likewise, spatial disorientation is among the earliest behavioral symptoms of Alzheimer’s disease and can lead to a loss of personal autonomy and heightened risk of mental distress, physical injury, or even death. Impaired spatial navigation has therefore gained traction as a promising diagnostic marker of age-related cognitive dysfunction and as a potential target for disease-modifying interventions (Coughlan et al., 2018; Segen et al., 2022).

Successful spatial navigation is typically conceived as relying on two complementary strategies. Allocentric or world-centered navigation requires an individual to learn the relationship between external spatial cues such as landmarks in order to form an internal topographic map of their environment. Egocentric or person-centered navigation strategies place a greater reliance on traversing familiar and well-learned routes, and are generally regarded as less flexible and efficient than allocentric navigation. The dominant theory in the field of aging is that allocentric-based navigation is impaired in older age, resulting in a preference for egocentric-based navigation strategies (Moffat et al., 2006; Moffat and Resnick, 2002).

Now, in eLife, Angelo Arleo and colleagues at Sorbonne Université – including Marcia Bécu as first author – report that the ability to engage in putative allocentric-based navigation may depend largely on the types of spatial cues present in the environment (Bécu et al., 2023). Critically, children and older individuals were just as likely as young adults to use allocentric strategies when geometric spatial cues (as opposed to landmarks) were available to guide navigation. The work adds to mounting evidence that allocentric navigation may show some degree of preservation with age.

Bécu et al. examined the types of strategies children, young adults, and healthy older adults used when navigating a Y-maze using a virtual reality headset that allowed them to move freely (Figure 1). The Y-maze has a long history of being used to examine spatial strategies in rodents. However, the task typically relies exclusively on visual landmarks to guide navigation. Here, participants were randomly assigned to navigate in one of two Y-Maze conditions. In the classic landmark condition, participants navigated an equiangular maze in which three distal landmarks surrounding the maze could be used to infer their location. In the geometry condition, the angles between respective maze arms were not equal, allowing participants to determine their position in the maze in the absence of landmarks. Children and older adults were more likely than young adults to prefer an egocentric strategy when navigating the landmark condition, replicating numerous prior studies. However, this age-related preference for egocentric navigation was eliminated in the geometry condition, with most participants in all three age groups preferring an allocentric search strategy.

**Figure 1.**
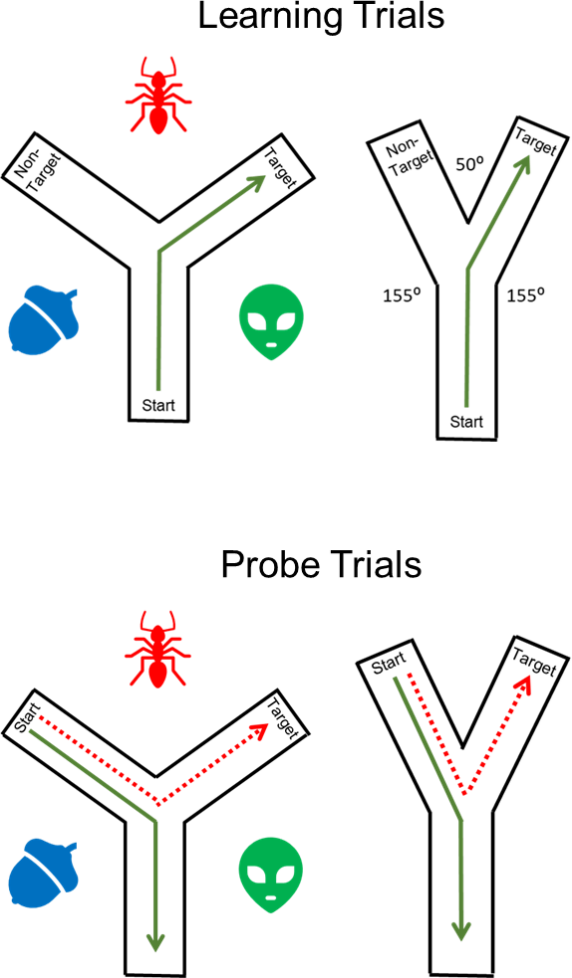
Exploring navigation strategies. In the Y-maze task, participants first take part in a learning trial (top). Participants are placed at the base of the Y-maze (start) in either the landmark condition (left) or the geometry condition (right). The landmark condition features a Y shape with equal angles between the arms and three landmarks surrounding the maze which are illustrated with blue, red and green shapes. There are no landmarks in the geometry condition: however, the angles in the Y-maze are not equal, and this is a geometric spatial cue that can be used for navigation. Participants are trained to locate the target arm by taking a specific turn at the maze junction (for example, a right turn in this example). The learned route is shown in green. During subsequent probe trials (bottom), participants are asked to locate the target from a new starting position (the non-target arm). The route taken in the probe trial reflects the navigation strategy used by the participant. Allocentric routes (dotted red lines) are those in which participants used the external landmarks or differences in maze angle to navigate to the target arm. Egocentric routes (green lines) are those in which participants repeat the previous learned response (for example, turn right at the junction), resulting in navigation to the non-target arm of the maze.

Next, Bécu et al. analyzed eye-tracking data to examine whether age differences in visual sampling of the environment might account for the age-related preference for egocentric navigation observed in the landmark condition. Young adults and older adults did not differ in the proportion of time spent visually fixating on the landmarks when learning the maze in the landmark condition. Likewise, there was no difference between those who used egocentric navigation and those who used allocentric navigation, independent of age. These results suggest that the age-related preference for egocentric navigation observed during the landmark condition was not caused by a failure to attend to the landmarks during learning. Instead, allocentric navigators, regardless of age, spent a greater proportion of time fixating on the landmarks as they planned a locomotor response during probe trials.

Taken together, the findings reported by Bécu et al. suggest that rather than a selective deficit in allocentric-based navigation, spatial challenges in older individuals may be the result of difficulties processing landmark cues in order to orient in space. These results complement recently published findings from a study of over 37,000 individuals collected using the mobile app Sea Hero Quest, which showed that landmark-based navigation strategies decline linearly with age (West et al., 2023). Critically, this work challenges decades of prior work to suggest that wayfinding deficits in older age are unlikely to be accounted for by a simple dichotomy between allocentric and egocentric navigation.

The finding that older adults seemingly maintain the ability to orient in space as well as their younger counterparts when geometric spatial cues are available offers exciting opportunities for future research. Studies examining the perceptual, cognitive and/or neural basis of geometric cue processing could make significant contributions to the development of more comprehensive models of aging and navigation. It will also be interesting to explore whether longitudinal declines in landmark-based processing are sensitive to pathologic changes associated with Alzheimer’s disease and related dementias. This knowledge could be important when designing accessible environments for vulnerable aging populations.

## References

[bib1] Bécu M, Sheynikhovich D, Ramanoël S, Tatur G, Ozier-Lafontaine A, Authié CN, Sahel J-A, Arleo A (2023). Landmark-based spatial navigation across the human lifespan. eLife.

[bib2] Coughlan G, Laczó J, Hort J, Minihane AM, Hornberger M (2018). Spatial navigation deficits - Overlooked cognitive marker for preclinical Alzheimer disease?. Nature Reviews Neurology.

[bib3] Head D, Isom M (2010). Age effects on wayfinding and route learning skills. Behavioural Brain Research.

[bib4] Levine TF, Allison SL, Stojanovic M, Fagan AM, Morris JC, Head D (2020). Spatial navigation ability predicts progression of dementia symptomatology. Alzheimer’s & Dementia.

[bib5] Moffat SD, Resnick SM (2002). Effects of age on virtual environment place navigation and allocentric cognitive mapping. Behavioral Neuroscience.

[bib6] Moffat SD, Elkins W, Resnick SM (2006). Age differences in the neural systems supporting human allocentric spatial navigation. Neurobiology of Aging.

[bib7] Segen V, Ying J, Morgan E, Brandon M, Wolbers T (2022). Path integration in normal aging and Alzheimer’s disease. Trends in Cognitive Sciences.

[bib8] West GL, Patai ZE, Coutrot A, Hornberger M, Bohbot VD, Spiers HJ (2023). Landmark-dependent navigation strategy declines across the human life-span: evidence from over 37,000 participants. Journal of Cognitive Neuroscience.

